# Connectivity of rapid-testing diagnostics and surveillance of infectious diseases

**DOI:** 10.2471/BLT.18.219691

**Published:** 2019-02-04

**Authors:** Damien Ming, Timothy Rawson, Sorawat Sangkaew, Jesus Rodriguez-Manzano, Pantelis Georgiou, Alison Holmes

**Affiliations:** a NIHR Health Protection Research Unit in Healthcare Associated Infections and Antimicrobial Resistance, Hammersmith Hospital Campus, London W12 0NN, England.; b Department of Electrical and Electronic Engineering, Imperial College, London, England.

The World Health Organization (WHO) developed the ASSURED criteria to describe the ideal characteristics for point-of-care testing in low-resource settings: affordable, sensitive, specific, user-friendly, rapid and robust, equipment-free and deliverable.^1^ These standards describe. Over the last decade, widespread adoption of point-of-care testing has led to significant changes in clinical decision-making processes. The development of compact molecular diagnostics, such as the GeneXpert® platform, have enabled short turnaround times and allowed profiling of antimicrobial resistance. Although modern assays have increased operational requirements, many devices are robust and can be operated within communities with minimal training. These new generation of rapid tests have bypassed barriers to care and enabled treatment to take place independently from central facilities. Here we describe the importance of connectivity, the automatic capture and sharing of patient healthcare data from testing, in the adoption and roll-out of rapid testing.

## Surveillance

Despite advances in technology, opportunities for rapid testing to provide further added value in health surveillance are still overlooked. Obtaining timely, patient-level data on emerging threats to global health, including antimicrobial resistance and infectious diseases leading to public health emergencies of international concern, is needed. Within fragile developing health systems, the availability of updated health data is essential. The use of decentralized rapid testing during outbreaks, such as the 2013–2016 Ebola virus disease outbreak, has been invaluable for improving triage, management and patient isolation. However, the disconnection between rapid testing and reporting led to difficulties in service planning and surveillance, as manually compiled patient line-lists were error-prone and often led to underreporting.^2^ Beyond epidemic settings, weak health reporting structures have also led to a dependence on sources of low-quality research data, resulting in biased or outdated estimates. Strategies to ensure good-quality information through active surveillance are costly and often poorly sustained. Promising approaches such as mobile health or using large datasets through machine learning are beginning to be implemented, although their roles within disease control programmes are yet to be defined.

The challenge of low quality and outdated surveillance data is directly relevant to global strategies tackling antimicrobial resistance. Many low-resource settings lack diagnostic laboratory capacity to support national surveillance and share data. In national tuberculosis programmes, district hospitals frequently rely on the Xpert® MTB/RIF to diagnose drug-resistant tuberculosis. However, in earlier systems, lack of an in-built, wireless function to transmit results data to third parties affected the ability to systematically capture patient data at programme level, as highlighted in an implementation study in Mozambique.^3^ Requirements for users of rapid testing devices to use proprietary reporting software might restrict the ability to transmit personal identifiable information, as manufacturer-owned servers may be subject to different data protection laws, depending on location. Stakeholders involved in healthcare need to explicitly address issues regarding responsibilities for secure data storage and authorized use of patient information. For data that is collected through rapid-testing by multiple agencies, including disease control programmes and nongovernmental organizations, data ownership can be difficult to define. Software systems also need to be sufficiently flexible to conform to data privacy laws, which differ across countries and software and could therefore lead to more errors. Initiatives such as the Global Antimicrobial Resistance Surveillance System (GLASS)^4^ aim to harmonize bacterial antimicrobial resistance data collection through common information and technology platforms. However, there is no open-source connectivity standard between manufacturers of rapid testing platforms.

## Health surveillance

Direct linkage of rapid-testing data and health surveillance has immense potential. Improved connectivity and the ability to directly capture testing data would fulfil important gaps in health surveillance. The significant increase in the use of rapid-testing should call for the update of guidelines, to explicitly emphasize requirements for connectivity.

Cellular communication networks are well established in most low- and middle-income countries and use of mobile phones is widespread and increasing. A flexible, ad hoc surveillance network could be established without significant additional infrastructure requirements, taking advantage of the geographical spread of rapid testing. Health facilities connected to central laboratories through rapid testing devices could automatically capture data on real-time disease frequency and health-care use according to location. Connectivity with supporting laboratories would also facilitate audits and quality assurance.^5^

Expanding connectivity would allow clinical decision support to use the same devices used for testing. A cloud-based artificial intelligence system could incorporate diverse sets of information including patient data, test results and up-to-date epidemiology, including drug-resistance profiles, to guide clinical treatment.^6^ This capacity would be important in settings where specialist input is unavailable and would support optimization of antimicrobial use.

For newer near-testing platforms, such as hand-held lab-on-a-chip devices capable of nucleic acid detection, direct secure data connection should be in-built. Test and geospatial data collected directly from mobile health workers could allow for real-time mapping of disease and for interventions to be rapidly focused. For existing point-of-care testing technologies like lateral flow tests, a system of direct reporting should be an integral part of the testing process. Incentive schemes and directed feedback to health-care workers have been successful in encouraging reporting of lateral flow test results for purposes of disease surveillance.^7^ The use of now widely-available technology such as mobile phone cameras to interpret and transmit images of malarial lateral flow tests has resulted in increased test sensitivity and reporting.^8^

## Real-time disease surveillance

**Fig. 1:**
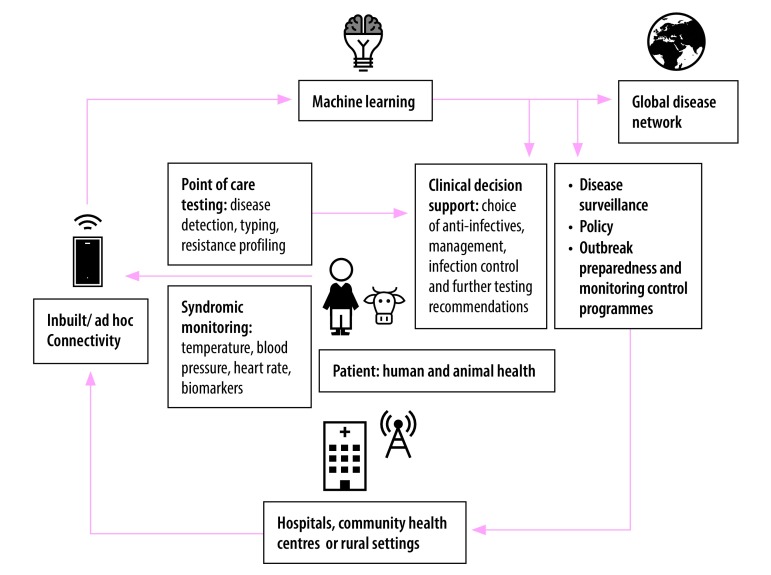
Integrated health surveillance system using existing cellular networks

Notes: This figure represents a proposed integrated health surveillance system that combines automatic reporting from rapid diagnostics and testing from human and animals across settings. The information is used locally to support clinical decision-making and is also automatically relayed through existing cellular networks or ad hoc connectivity using mobile phones. Data are combined with syndromic surveillance information to generate outbreak alerts through machine learning. The integration of information from different sources or testing sites can be used to inform clinical management, antimicrobial policy, disease surveillance as well as areas with potential disease outbreaks.

Connectivity in diagnostics is a prerequisite for the establishment of a robust system of real-time disease modelling and epidemic forecasting. The provision of direct patient-level data in the context of existing global surveillance systems through WHO would serve as a platform to enhance prediction of major disease outbreaks. There is also increasing interest in developing low cost minimally-invasive biosensors capable of recording and transmitting physiological indices and biomarkers.^9^ Including this additional layer of syndromic surveillance to real-time disease forecasting could enhance performance in the early detection of disease onset in a susceptible population. Targeted control interventions, including vector control and health resource mobilization, could be deployed before an outbreak (Fig. 1). The use of real-time epidemiological data would strengthen strategies such as ring vaccination. Using biosensors for surveillance would be particularly suited for animal health, where monitoring of large-scale farming environments or migratory populations would be useful for zoonotic outbreaks and impact on human health care. Although we primarily focus on humans, ensuring that diagnostic development priorities and methods of antimicrobial surveillance are in line the One Health strategy will be equally important.

## Future challenges

Data linkage needs to be adapted to existing reporting systems and ensure that information is transmitted securely. Patient consent for information use and a right to privacy will need to be established, particularly in scenarios that involve self-testing. Rapid-testing platforms have been developed for tuberculosis and human immunodeficiency virus because of global burden and available funding. However, the results of progress made in developing rapid testing platforms and technological solutions could be transferable to other diseases. Innovative techniques, such as microfluidics and semiconductor arrays that detect nucleic amplification, are currently undergoing validation. Platforms for antimicrobial resistance surveillance^10^ and detection of dengue serotypes directly from blood samples^11^ will enter clinical trials in the next five years. Therefore, direct applications of rapid testing platforms shaping clinical practice, public health policy and research priorities in the next 10 years are possible. The lower cost of electronic manufacturing and emerging market demands in low- and middle-income countries, which bear a disproportionate burden of infectious diseases, are an impetus to ongoing development and widespread roll-out of such platforms.

## A proposal for connectivity 

Here we propose a basic set of connectivity standards that should be included in target product profiles for rapid-testing. First, all future development of rapid-testing platforms must explicitly address the need for data connectivity and reporting. Devices should preferably be capable of wireless communication through existing cellular networks without additional requirements for setup. Second, information exchange should take place using a common, secure protocol that is open-source and independent of manufacturer. This information exchange needs to be flexible to retrofit existing testing systems and adapt to other sources of data input, for example, use of mobile phone camera images to upload lateral flow test results. Third, the information network should cover a wide geographical area and coordinate with local cellular network companies. Information needs to be accessible securely through devices such as phone, basic computers and mobile apps. These connectivity requirements should be developed closely alongside WHO’s essential diagnostics list.

Establishing connectivity standards will aid national surveillance of notifiable infections through enabling automatic reporting and improving data completeness. Although the current panel of diseases and performance of rapid tests available are currently limited, the role of rapid testing is likely to increase significantly. Therefore, establishing a connectivity framework is needed. Studies support the cost–effectiveness of widespread rapid-testing system for tuberculosis,^12^ although further analyses should be carried out for other diseases and settings. The improved transparency and accountability resulting from information-sharing across a network could also improve timeliness of notification of disease outbreaks. The ability to identify disease outbreaks promptly and capture accurate information through this network would enable additional measures to be activated, such as access to emergency funding, which will be important for control interventions.

Seizing the opportunity to harness detailed, real-time data directly from rapid-testing processes would bolster disease surveillance and epidemic preparedness, but will need careful early coordination between manufacturers, stakeholders and international health bodies. 
